# Fibroblast Activation Protein Alpha (FAP) Expression Is Associated with Disease Recurrence and Poor Response to Tyrosine Kinase Inhibitors in Advanced Clear Cell Renal Cell Carcinoma

**DOI:** 10.3390/ijms262211112

**Published:** 2025-11-17

**Authors:** María Riaza Montes, Beatriz Suárez, Jon Danel Solano-Iturri, David Lecumberri, Ane Miren Iturregui, Charles H. Lawrie, María Armesto, Caroline E. Nunes-Xavier, Rafael Pulido, José I. López, Javier C. Angulo, Gorka Larrinaga

**Affiliations:** 1 Department of Urology, Galdakao-Usansolo University Hospital, 48960 Usansolo, Spain; maria.riazamontes@osakidetza.eus; 2 Biobizkaia Health Research Institute, 48903 Barakaldo, Spain; jondanel.solanoiturri@osakidetza.eus (J.D.S.-I.); carolineelisabeth.nunes-xavier@bio-bizkaia.eus (C.E.N.-X.); rpulidomurillo@gmail.com (R.P.); jilpath@gmail.com (J.I.L.); 3 Department of Nursing, Faculty of Medicine and Nursing, University of the Basque Country (UPV/EHU), 48940 Leioa, Spain; beatriz.suarez@ehu.eus; 4 Pathology Department, Cruces University Hospital, 48903 Barakaldo, Spain; 5 Department of Urology, Cruces University Hospital, 48903 Barakaldo, Spain; david.lecumberricastanos@osakidetza.eus (D.L.); anemiren.iturreguidelpozo@osakidetza.eus (A.M.I.); 6 Molecular Oncology Group, Biogipuzkoa Health Research Institute, 20014 San Sebastián, Spain; charles.lawrie@bio-gipuzkoa.eus (C.H.L.); maria.armestoalvarez@bio-gipuzkoa.eus (M.A.); 7 IKERBASQUE, Basque Foundation for Science, 48009 Bilbao, Spain; 8 Radcliffe Department of Medicine, University of Oxford, Oxford OX3 9DU, UK; 9 Sino-Swiss Institute of Advanced Technology (SSIAT), Shanghai University, Shanghai 201800, China; 10 Department of Tumor Biology, Institute for Cancer Research, Oslo University Hospital Radiumhospitalet, 0424 Oslo, Norway; 11 Department of Urology, Hospital Universitario de Getafe, 28907 Getafe, Spain; javier.angulo@salud.madrid.org; 12 Faculty of Biomedical and Health Sciences, Department of Medicine, Universidad Europea de Madrid, 28670 Madrid, Spain; 13 Department of Physiology, Faculty of Medicine and Nursing, University of the Basque Country (UPV/EHU), 48940 Leioa, Spain

**Keywords:** clear cell renal cell carcinoma, metastasis, disease recurrence, fibroblast activation protein, FAP, survival, tyrosine kinase inhibitors

## Abstract

Despite advances in the management of advanced clear cell renal cell carcinoma (ccRCC), robust biomarkers for prognosis and therapeutic response prediction remain elusive. Fibroblast activation protein-α (FAP), a marker of activated cancer-associated fibroblasts (CAFs), has emerged as a potential indicator of tumor aggressiveness and resistance to systemic therapies in various solid tumors. This study evaluated the clinical relevance of stromal FAP expression in a cohort of 137 patients with advanced ccRCC and long-term follow-up. FAP immunohistochemistry (IHC) was performed on primary tumor specimens and correlated with key clinicopathological features, disease-free survival (DFS), overall survival (OS), and radiological response to first-line tyrosine kinase inhibitors (TKIs). A significantly higher percentage of FAP-positive CAFs was observed in primary tumors with high histological grade, extensive local invasion (pT3–4), and advanced clinical stage (NCCN stage III–IV). Stromal FAP expression was associated with shorter DFS and OS. Moreover, tumors lacking FAP expression were more likely to achieve complete response to TKI therapy as defined by RECIST criteria. These findings highlight the potential of FAP IHC as a prognostic and predictive tool in advanced ccRCC and support further clinical validation.

## 1. Introduction

Clear cell renal cell carcinoma (ccRCC) is the most common histological subtype of kidney cancer, accounting for approximately 70–80% of all renal malignancies [1]. According to GLOBOCAN data [2], kidney cancer was responsible for an estimated 434,419 new cases and 155,702 deaths worldwide in 2022 alone, highlighting its significant global health burden. The clinical impact of ccRCC is further compounded by its frequent presentation at advanced stages: approximately 30% of patients present with synchronous metastases, while an additional 30% of those with initially localized disease eventually develop distant metastases. [3]. Despite improvements in imaging, surgical techniques, and systemic therapies, metastatic ccRCC remains a formidable clinical challenge, with 5-year survival rates below 15% in patients presenting with widespread metastases [1,4].

The therapeutic landscape of advanced ccRCC has evolved significantly in recent years, largely driven by the advent of tyrosine kinase inhibitors (TKIs) and the incorporation of immune checkpoint inhibitors (ICIs) [5,6]. Combination regimens, either ICI–ICI or ICI–TKI, are now established as standard first-line options, tailored according to individual patient risk profiles. Nevertheless, therapeutic responses remain highly heterogeneous, and a substantial proportion of patients exhibit either primary resistance or limited clinical benefit [1,5,6].

This variability continues to challenge current treatment algorithms and exposes the limitations of existing prognostic tools [1,4]. Risk models such as the International Metastatic Renal Cell Carcinoma Database Consortium (IMDC) classification provide valuable clinical guidance but lack integration of molecular or tumor-intrinsic features [7]. Although numerous molecular and immune-based biomarkers have shown potential in retrospective or exploratory studies, none have yet reached clinical implementation for patient stratification or treatment selection [8].

Within this evolving therapeutic context, increasing attention has turned to the tumor stroma and, in particular, cancer-associated fibroblasts (CAFs) as essential contributors to the ccRCC tumor ecosystem [9]. CAFs are among the most abundant non-neoplastic components of the tumor microenvironment (TME) and are recognized as active architects of tumor progression [10,11]. Through dynamic crosstalk with malignant and other stromal cells, CAFs regulate critical processes such as epithelial–mesenchymal transition, extracellular matrix remodeling, and the establishment of immunosuppressive niches. Together, these functions contribute to tumor growth, invasiveness, and therapeutic resistance [9,11]. Consequently, CAF-associated markers have emerged as promising targets for diagnostic refinement and therapeutic intervention.

Fibroblast activation protein-α (FAP), a membrane-bound serine protease, is one of the most studied CAF markers and plays a central role in tumor–stroma interactions [12]. While not yet used in routine clinical practice, FAP expression has been associated across several solid tumors with greater tumor aggressiveness and worse survival outcomes [12,13]. Its selective expression in tumor-associated stroma and minimal presence in normal adult tissues further underscores its diagnostic specificity. These features, along with emerging data from early-phase clinical trials, support its development as a therapeutic target and as a candidate for molecular imaging [12,14,15].

Our own experience, and that of other groups, support these observations in renal neoplasia. FAP is expressed in the stroma of the three most common malignant renal tumors—clear cell, papillary, and chromophobe RCC—but not in renal oncocytoma, suggesting a link with malignancy and invasiveness [16]. In ccRCC specifically, stromal FAP expression correlates with adverse pathological features and poorer survival [16,17,18]. Moreover, high *FAP* mRNA levels have been associated with increased risk of recurrence and poor response to TKIs and ICIs [19,20], supporting its role as a potential prognostic and predictive biomarker in advanced disease.

Based on this background, the present study was designed with two main objectives: first, to assess whether FAP protein expression, as evaluated by immunohistochemistry (IHC), predicts disease recurrence in patients with apparently localized ccRCC who subsequently developed distant metastases; and second, to investigate the association between stromal FAP expression in primary tumors and clinical response to first-line TKI therapy in patients with advanced ccRCC.

## 2. Results

### 2.1. Clinical and Pathological Characteristics of ccRCC Patients and Samples

A well-characterized cohort of 137 patients with advanced ccRCC [21] was included in the study. The mean age was 59.33 years (median 60 years, and range: 26–83 years). Male-to-female ratio was 2.42:1 (male and female mean age was 59.15 and 59.75 years, respectively). The average diameter of the primary tumors was 8.73 cm, with a median size of 8 cm. Fuhrman nuclear grade was distributed as follows: grade 1 in 6 cases (4.4%), grade 2 in 26 (19%), grade 3 in 43 (31.4%), and grade 4 in 62 patients (45.3%). According to the AJCC pathological T classification [22], 22 tumors (16.1%) were staged as pT1, 15 (10.9%) as pT2, 91 (66.4%) as pT3, and 9 (6.6%) as pT4. At the time of nephrectomy, staging based on the NCCN criteria revealed that 17 patients (12.4%) had stage I disease, 10 (7.3%) stage II, 48 (35%) stage III, and 62 (45.3%) stage IV.

Nodal involvement was confirmed in 22 patients (16%), with 15 (10.9%) classified as N1 (single lymph node affected) and 7 (5.1%) as N2 (multiple nodes), whereas 115 cases (83.9%) showed no lymphatic metastasis (N0). The cohort included both patients who were diagnosed with synchronous distant metastases (M1; *n* = 55, 40.1%) and those initially staged as M0 (*n* = 82, 59.9%) who subsequently developed metachronous metastases during follow-up.

Functional status at diagnosis, assessed via the Eastern Cooperative Oncology Group (ECOG) scale [23], indicated that 113 patients (82.5%) were fully active (ECOG 0), 21 (15.3%) had slight activity restrictions (ECOG 1), and 3 (2.2%) showed more marked limitations (ECOG 2). Risk stratification using the International Metastatic Renal Cell Carcinoma Database Consortium (IMDC) criteria [7] categorized 59 patients (43.1%) as favorable risk, 66 (48.2%) as intermediate, and 12 (8.8%) as poor risk.

All clinical and pathological characteristics are summarized in Table 1. For statistical purposes, prognostic variables were grouped and dichotomized when appropriate, enabling clearer comparisons and more robust interpretability across subgroups.

### 2.2. FAP Expression in Primary ccRCCs in Relation to Aggressive Pathological Features

We analyzed FAP expression in primary tumor tissue according to key pathological indicators of tumor aggressiveness, including Fuhrman nuclear grade, tumor size, local extension (pT), nodal involvement (N), presence of distant metastases, NCCN stage, ECOG performance status, and IMDC risk classification. For statistical purposes, these variables were dichotomized as shown in Table 1, to facilitate comparisons and enhance interpretability.

**Figure 1:**
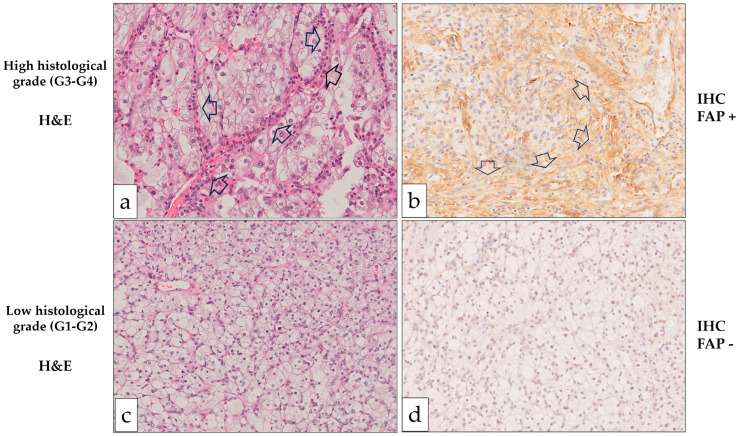
Hematoxylin/Eosin (H&E) and FAP immunohistochemical (IHC) staining in clear cell renal cell carcinoma (ccRCC). (**a**,**b**): High-grade ccRCC with prominent stromal proliferation (arrows) separating tumor cell nests showing prominent FAP positivity in stromal fibroblasts (arrows). (**c**,**d**): Low-grade CCRCC without apparent stromal proliferation and FAP negative staining. (Original magnification ×250).

Following the same protocol as our previous studies [16,18], FAP was considered positive when strong cytoplasmic immunostaining was detected in tumor interstitial cells previously identified as CAFs in H&E sections (Figure 1).

#### 2.2.1. FAP Expression Is Higher in High-Grade Primary ccRCCs

**Figure 2:**
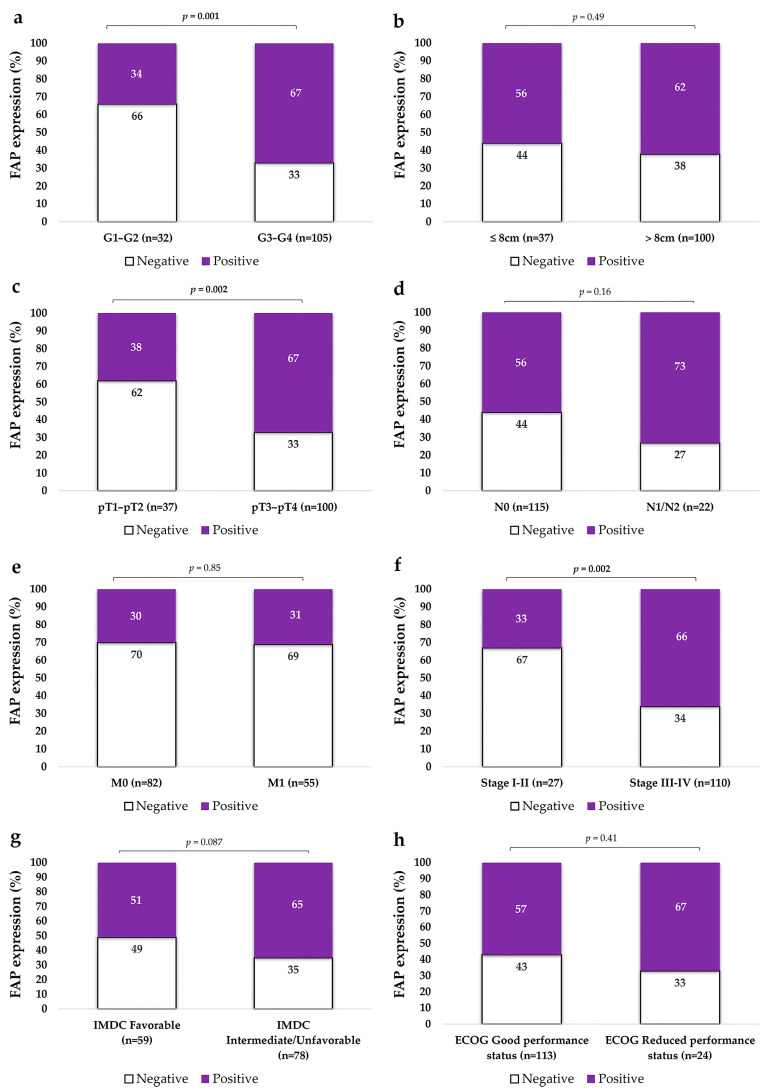
Immunohistochemical expression of FAP in primary ccRCC tumors stratified by clinicopathological variables. Tumors were grouped according to histological grade (**a**), tumor size (**b**), local invasion (pT) (**c**), lymph node involvement (N) (**d**), presence of distant metastases (M) (**e**), and NCCN stage at diagnosis (**f**). IMDC risk classification (**g**) and ECOG performance status (**h**) were assessed at the time of initiating TKI therapy. FAP expression in CAFs was categorized as positive or negative. The Y-axis represents the percentage of tumors exhibiting FAP positivity or negativity. Statistical comparisons were performed using the χ^2^ test. N0: No lymph node metastasis; N1: lymph node metastasis; M0: No distant metastasis; M1: synchronous distant metastasis.

Fuhrman histological grade of primary tumors was categorized into low (G1–G2) and high grades (G3–G4). FAP-positive CAFs were significantly more frequent in the stroma of high-grade ccRCCs compared to low-grade ones, as illustrated in Figure 1 and Figure 2 (Figure 2a).

#### 2.2.2. FAP Expression Is Higher in Non-Organ Confined Tumors

Given that FAP IHC expression was analyzed as a binary variable (positive vs. negative), tumor size—a continuous variable—was transformed into categorical groups to enable statistical comparison using the chi-square test. Two stratification approaches were applied: first, a dichotomization based on the median tumor diameter in our series (8 cm); and second, cut-off points commonly used in the pT classification (4 cm, 7 cm, and 10 cm). No significant association was observed between FAP expression and tumor size when using the median cut-off (Figure 2b). Likewise, no statistically significant differences were found when applying pT-based thresholds at 4 cm (χ^2^, *p* = 0.63), 7 cm (χ^2^, *p* = 0.47), or 10 cm (χ^2^, *p* = 0.87).

Furthermore, when results were stratified according to local invasion (pT), FAP expression was significantly more frequent in non–organ-confined tumors (pT3–pT4) compared to organ-confined tumors (pT1–pT2) (Figure 2c).

#### 2.2.3. FAP Expression Is Higher in Tumors with Advanced Stage and Metastatic Spread

The proportion of FAP-positive (FAP+) tumors was higher among cases with locoregional lymph node metastases (73%) compared with those without nodal involvement (53%), although this difference did not reach statistical significance (*p* = 0.16; Figure 2d). All primary tumors in the series eventually developed distant metastases; nevertheless, we compared FAP expression between patients who presented with metastases and those who did not at initial diagnosis. No differences were observed between both groups (Figure 2e). In contrast, stratification according to the 2010 NCCN staging system [22] revealed that stage III–IV tumors exhibited significantly higher FAP expression than stage I–II tumors (*p* < 0.05; Figure 2f).

#### 2.2.4. FAP Expression Does Not Significantly Vary According to IMDC Risk Classification or ECOG Performance Status

When stratified by IMDC risk classification, a decreasing trend in FAP expression was observed in tumors from patients with favorable prognosis compared to those with intermediate or poor prognosis, although the difference was not statistically significant (Figure 2g). Regarding functional status, FAP expression was similar in tumors from patients with mildly to moderately limited performance (ECOG 1–2) and those with fully preserved function (ECOG 0) (Figure 2h).

### 2.3. FAP Expression in the Primary Tumor According to Treatment Response

Fifty-five patients of the series presented synchronous metastases and were treated with adjuvant TKIs after surgery. The remaining 82 patients were initially disease-free following nephrectomy but developed metastases months or years after surgery. As a result, the entire cohort of 137 patients received first-line TKI-based therapy with sunitinib, sorafenib, or pazopanib.

Treatment response was evaluated using both the RECIST [24] and MASS [25] criteria. Based on the RECIST classification, 57 patients (41.6%) experienced disease progression, 16 (11.7%) achieved a complete response, 32 (23.4%) had a partial response, and 31 (22.6%) exhibited stable disease. MASS-based evaluation yielded comparable results: 58 patients (42.3%) showed an unfavorable response, 45 (32.8%) had a favorable response, and 34 (24.8%) were classified as indeterminate.

#### FAP-Positive Tumors Were Less Responsive to Treatment

**Figure 3:**
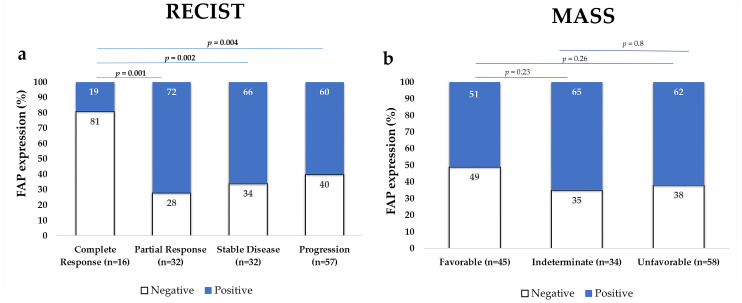
Immunohistochemical expression of FAP in primary ccRCC tumors according to response to TKI therapy, evaluated using RECIST criteria (**a**) and MASS criteria (**b**). The percentage of FAP-positive tumors was significantly lower among cases with complete response according to RECIST. The Y-axis indicates the percentage of tumors classified as FAP positive or negative. Statistical analysis was performed using the χ^2^ test.

According to RECIST criteria, only 19% of tumors that achieved a complete response showed FAP positivity in the stromal compartment, while 60–72% of those with partial response, stable disease, or progression were FAP positive (χ^2^, *p* = 0.004). In contrast, when applying the MASS criteria, FAP positivity was more frequent in tumors with indeterminate or unfavorable responses than in those with favorable response, although the difference was not statistically significant. These findings are illustrated in Figure 3.

### 2.4. FAP Expression in ccRCC According to Patients’ Disease-Free (DFS) and Overall Survival (OS)

DFS was defined as the time between nephrectomy and the initiation of first-line TKI therapy, which was administered to all patients shortly after recurrence diagnosis. This variable reflects the duration of remission following initial curative-intent surgery [26]. In this cohort, DFS was analyzed over a 10-year period, as the vast majority of patients who were disease-free at the time of nephrectomy experienced metastatic relapse within that timeframe. Indeed, metastases were diagnosed at a mean of 39.0 months (median: 20.5), and by 10 years, 76 of the 82 patients (92.7%) had relapsed. To ensure consistent follow-up and comparability across groups, patients who remained disease-free beyond 120 months were administratively censored at that point.

OS was defined as the time from diagnosis to death from any cause [27]. It was assessed over a 15-year period to accommodate the longer follow-up required to capture mortality events in this cohort. Accordingly, the mean follow-up for the 137 patients included in the study was 66.9 months (median: 49.0), and by 15 years, 129 patients (94.2%) had died, while 8 (5.8%) were still alive.

#### 2.4.1. FAP Expression Is Associated with Shorter DFS in ccRCC Patients

**Figure 4:**
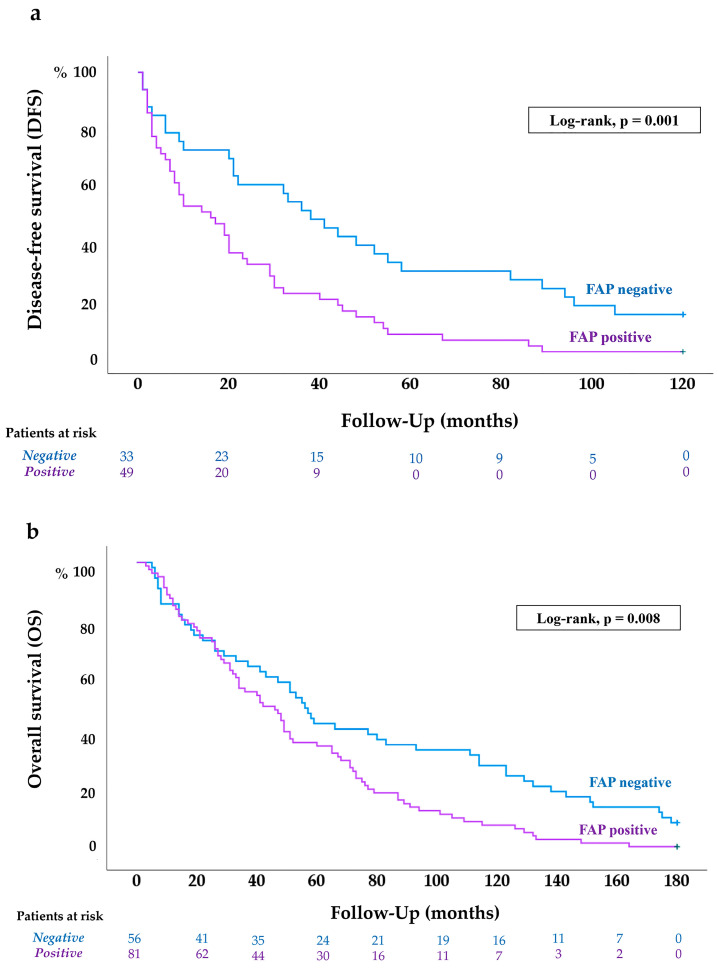
FAP expression in primary ccRCC tumors and patient survival. Kaplan–Meier curves and log-rank tests show a significant association between FAP positivity and DFS at 10 years (**a**), as well as OS at 15 years (**b**). The number of patients at risk at specific time points is shown below each curve.

Kaplan–Meier survival curves and the log-rank test (*p* = 0.001) revealed that FAP expression in CAFs of primary ccRCCs was significantly associated with an increased risk of metastatic recurrence following nephrectomy (Figure 4a).

Univariate analysis of 10-year DFS is summarized in Table 2. Certain variables were excluded from this analysis: metastasis status (M0 in all cases, as the subgroup comprised only metachronous patients), NCCN stage (due to its overlap with pT and N categories), and MASS and RECIST scores (as they reflect treatment response, and these patients had not yet received systemic therapy). Similarly, ECOG performance status and IMDC risk score were not considered, as both were assessed at the time of recurrence and treatment initiation.

Variables analyzed included age, histological grade, pT stage, N, and FAP expression. All variables were categorized into binary groups, as outlined in Table 1, to facilitate robust statistical comparison and clearer interpretability. All four variables showed significant associations with DFS and were therefore entered into a multivariate Cox regression model. However, using a backward stepwise (Wald) method, FAP expression did not retain independent prognostic value, whereas age, tumor grade, and pT stage remained statistically significant predictors of disease-free survival (Table 2).

#### 2.4.2. FAP Expression Is Associated with Worse OS of ccRCC Patients

Kaplan–Meier survival analysis and the log-rank test (*p* = 0.008) revealed that FAP expression in CAFs from primary ccRCCs was significantly associated with reduced overall survival (OS) (Figure 4b).

Subsequently we undertook univariate Cox regression analysis to assess the relationship between OS and a range of clinical and pathological variables over a 15-year follow-up period (Table 2). The analysis identified age, histological grade, pT stage, lymph node involvement (N), distant metastasis at diagnosis (M), ECOG performance status, IMDC risk classification, MASS score, RECIST response, and FAP expression as significantly associated with overall survival. These variables were subsequently included in the multivariate Cox regression model.

To avoid redundancy due to collinearity, tumor diameter was excluded, as it is encompassed by the pT stage. NCCN 2010 stage was also omitted given its composite nature, integrating pT, N, and M categories.

In the multivariate analysis, conducted using a backward stepwise (Wald) elimination method, age, pT stage, N, M, and MASS score emerged as independent prognostic factors for 15-year OS. FAP expression in the primary tumor, however, did not maintain statistical significance as an independent predictor (Table 2).

### 2.5. FAP mRNA Expression in ccRCC (In Silico Analysis)

In order to increase the clinical significance of our findings we carried out in silico analyses of *FAP* mRNA expression in 533 ccRCC cases using the TCGA database. Consistent with our experimental data we confirmed that indeed higher *FAP* expression was observed in tumour tissue compared with normal kidney tissue. Furthermore, we also observed that high *FAP* expression correlated with lower overall survival, and with higher TNM stage and pathologic stage (pT) (*p* < 0.0001 in all cases) (Supplementary Figure S1).

## 3. Discussion

This study aimed to evaluate the clinical relevance of FAP expression in CAFs from primary tumors of patients with advanced ccRCC. By analyzing a well-characterized cohort of synchronous and metachronous metastatic cases treated with TKIs, we confirmed that stromal FAP expression is significantly associated with pathological features of aggressive disease, such as higher tumor stage and histological grade. Moreover, we investigated whether IHC detection of FAP could serve as a prognostic biomarker for disease recurrence and OS, as well as a predictor of treatment response. The study explores its potential clinical utility in risk stratification and therapeutic decision-making in advanced ccRCC.

ccRCC is characterized by its unpredictable clinical course, with approximately one-third of patients presenting with synchronous metastatic disease at diagnosis and another third developing distant metastases months or even years after curative-intent nephrectomy [1,3]. In previous studies, we have demonstrated that FAP expression in CAFs from primary ccRCC tumors is significantly higher in synchronous metastatic cases (M1) compared to those without metastases (M0) after long-term follow-up [16,17,28]. Importantly, FAP expression was also found to be significantly elevated in M0 tumors that eventually developed metachronous metastases [16]. It suggests that FAP IHC could serve as a prognostic tool to identify high-risk patients within the apparently localized M0 subgroup.

To explore this hypothesis, the present study included a well-defined cohort of 82 patients who were metastasis-free following nephrectomy but later developed metachronous metastases, with a follow-up period extending beyond 10 years. Univariate analysis demonstrated that FAP expression in CAFs was significantly associated with reduced DFS, consistent with its potential role in facilitating tumor recurrence. Furthermore, Ambrosetti et al. demonstrated this association at the mRNA level in both their independent clinical cohort and the TCGA dataset [19], consistent with our own TCGA results. Collectively, these data reinforce the hypothesis that FAP expression in the tumor stroma may enhance the likelihood of distant dissemination in ccRCC.

In line with earlier reports [16,17], we observed that FAP positivity in primary tumors was associated with reduced OS over a 15-year follow-up. However, this association did not remain significant in the multivariate model, where classical pathological variables such as pT stage, nodal involvement (N), and synchronous metastasis (M1) prevailed as independent predictors. Notably, our own previous studies have also yielded mixed results regarding the independent prognostic value of FAP [16,17,28], suggesting that its contribution may vary depending on cohort composition, modeling complexity and disease stage.

Our current cohort was intentionally enriched with advanced-stage cases, including both synchronous and metachronous metastases, selected to investigate biomarkers of response to TKIs, as previously reported [21]. Consequently, patients who remained metastasis-free after surgery during more than 10 years of follow-up were not included, limiting the generalizability of our findings to the broader ccRCC population. While this design complements our earlier research in localized tumors [16], it introduces a degree of selection bias that must be mentioned. Nevertheless, the consistent univariate association between FAP expression and both DFS and OS reinforces its relevance as a prognostic marker within the tumor stroma, even if not retained as an independent factor in multivariate modeling.

A particularly robust and reproducible finding across studies in ccRCC [16,17,28] and other epithelial tumors [13] is the association between FAP expression and local tumor invasion. Tumors with high pT stage consistently exhibit elevated FAP levels, supporting the notion that FAP + CAF-rich microenvironments contribute to an invasive phenotype. Given that pT stage is an independent predictor of both DFS and OS, the prognostic value of FAP may partly reflect its involvement in promoting local invasion. Indeed, rather than viewing FAP expression as a surrogate marker, its presence might directly influence tumor behavior through mechanisms that facilitate tumor growth and tissue infiltration. Therefore, the lack of independent significance in multivariate analysis should not be interpreted as a limitation, but rather as evidence that FAP expression is biologically intertwined with a key histopathological determinant of aggressiveness such as local invasion, which dominate prognostic modeling.

The proliferative and invasive capabilities of ccRCC primary tumors are critically shaped by the functional reprogramming of FAP + CAFs and the stromal remodeling they induce [29]. FAP modulates the extracellular matrix through both enzymatic degradation and non-enzymatic scaffolding effects, contributing to increased tissue stiffness and tumor invasiveness [9,11]. Moreover, FAP + CAFs secrete a variety of cytokines and chemokines that orchestrate an immunosuppressive TME [11,29]. This includes the recruitment and polarization of M2-like tumor-associated macrophages and the expansion of regulatory T cells (Tregs), both of which have been previously linked to adverse clinical outcomes in ccRCC [29,30,31]. In fact, we recently demonstrated, using multiplex immunofluorescence, that the co-localization of FAP + CAFs with these immune cells within the tumor microenvironment was independently associated with reduced survival [28], reinforcing the pathogenic synergy among these components.

The link between FAP expression and therapeutic response further supports this integrative role. Consistent with transcriptomic evidence [19,20], our study found that FAP-negative tumors were more likely to achieve a complete response (CR) to TKI therapy according to RECIST criteria. FAP + CAFs have been increasingly implicated in mechanisms of therapeutic resistance across solid tumors, including ccRCC [11,29]. These stromal cells can create a physical barrier to drug delivery and release pro-survival factors—such as interleukin-6 and stromal-derived factor-1—that modulate tumor cell plasticity and sustain a proangiogenic milieu counteracting VEGF-targeted inhibition [11,29]. Collectively, these data indicate that FAP expression delineates a tumor microenvironment that is both pro-invasive and treatment-refractory.

When treatment response was stratified by the MASS score, the association did not reach statistical significance. This discrepancy may reflect fundamental differences between the two scoring systems. RECIST focuses on quantifiable reductions in tumor size [24] and generates a binary, easily interpretable classification of response. In contrast, MASS integrates additional parameters such as changes in attenuation, morphology, and structure [25], potentially capturing earlier or heterogeneous treatment effects that may be less directly linked to baseline stromal features like FAP expression. Additionally, the larger proportion of “indeterminate” responses defined by MASS may obscure more subtler group-level differences.

A clearer understanding of how FAP + CAFs modulate the TME is crucial for designing more effective therapeutic combinations. From a translational perspective, FAP + CAFs are emerging as promising targets to improve the efficacy of systemic therapies. Preclinical and early-phase clinical studies in other tumor types have evaluated FAP-directed approaches—including monoclonal antibodies, CAR-T cells, and enzymatic inhibitors—with encouraging outcomes [29]. In ccRCC, where resistance to TKIs and ICIs remains a major barrier to durable disease control [5,6], combining these agents with FAP-targeted therapies may provide a rational strategy to overcome stromal-mediated resistance. Moreover, FAP IHC could help identify patient subsets most likely to achieve sustained responses or to benefit from stromal-directed interventions. Future research should determine whether integrating FAP expression into current prognostic models enhances clinical decision-making in advanced ccRCC.

## 4. Materials and Methods

### 4.1. Patients

This retrospective study included patients with advanced ccRCC treated between 2008 and 2018 at two tertiary hospitals in the Basque Country (Spain): Donostia and Cruces University Hospitals. All patients underwent radical nephrectomy, which confirmed the diagnosis of ccRCC. All patients eventually developed metastatic disease, either at the time of diagnosis or during follow-up. Metastases were classified as synchronous or metachronous based on radiological imaging, occasionally supplemented by biopsy or surgical resection. Tumors were staged at the time of nephrectomy according to the AJCC and NCCN 2010 systems [22].

Clinical performance was assessed using the ECOG scale [23] at the start of systemic treatment, and patients were stratified into prognostic categories using the IMDC classification system [7]. All included patients received first-line monotherapy with TKIs, continuing treatment until progression or loss of efficacy. Tumor response was assessed three months after treatment initiation using both RECIST [24] and MASS [25] criteria. The study protocol was approved by the Institutional Ethics Committee (CEIm Euskadi, PI2015059X, approved on 5 December 2022, Vitoria-Gasteiz, Spain).

The initial study population consisted of a previously reported cohort of 170 ccRCC patients treated with TKIs [21]. Twenty-five patients were excluded due to undefined treatment response (*n* = 5), non-clear cell histology (*n* = 4), or loss to follow-up (*n* = 16). Additionally, tissue microarrays (TMAs) from eight patients were excluded from the analysis due to absent tumor cores or technical artifacts. The final cohort included 137 evaluable patients.

### 4.2. Immunohistochemical Detection of FAP in ccRCC Samples

FAP was immunodetected in formalin-fixed, paraffin-embedded (FFPE) tissue sections using a rabbit monoclonal antibody (Ab207178, Abcam; lot: GR3374920-9, Cambridge, UK) at a 1:100 dilution [28]. Immunostaining was performed using the Dako Autostainer Plus (Dako Autostainer Plus, Dako, Santa Clara, CA, USA) according to standard protocols. Antigen retrieval was carried out by incubating the sections at 95 °C for 20 min in a high-pH retrieval buffer (K8005, Dako, Santa Clara, CA, USA). After rinsing, slides were incubated with the primary antibody for 50 min at room temperature. Subsequently, samples were incubated with an HRP-conjugated secondary anti-rabbit antibody (K8021, Dako) for 20 min. Detection was performed using the EnVision-Flex system (SM802, Dako), and peroxidase activity was visualized with diaminobenzidine (DAB; DM827, Dako). Sections were counterstained with hematoxylin (K8008, Dako) and mounted for evaluation.

Tissue microarrays (TMAs) were constructed using primary ccRCC samples, including two cores per tumor, and two cores of non-neoplastic kidney tissue as internal negative controls. ccRCC is a paradigmatic example of inter and intratumor heterogeneity [4,30]. As a result, the same tumor may contain, for example, typical low-grade clear cells in a region and high-grade undifferentiated cells with eosinophilic cytoplasm in another one. Also, the spectrum of histologies, grades, and the amount of non-tumor interstitial cells (lymphocytes, macrophages, fibroblasts…) between different tumors is very high and unpredictable. This variability cannot be avoided in any histological study and must be taken into account when building TMAs for immunohistochemical analysis.

As a consequence, semi-quantitative scoring methods (e.g., low/medium/high) were deemed unreliable by pathologists, given the variability in stromal fibroblast content across different areas of the same tumor. Therefore, FAP expression was recorded qualitatively as either present (+) or absent (−) in the stromal fibroblasts adjacent to tumor nests. To ensure greater consistency in histological evaluation, a sample was considered positive when FAP immunostaining was present, even if this is minimal (restricted to isolated cells), and negative if no FAP immunostaining was seen at all across the sample. We have considered that the quantification of the percentage of FAP positive cells with respect to the total non-tumor cells in such a small fragment of tissue (2.5 mm in diameter cores) may introduce unnecessary bias in the analysis. For this reason, we favored the simplicity in the evaluation to strengthen intra and inter-observer reproducibility. Two independent observers (JDSI and JIL) blindly evaluated all the samples; in the isolated cases of discrepancy, the slides were re-reviewed under a multi-head microscope to reach a consensus. If one of the two cores of each case in the series showed FAP positive CAFs, the case was classified as positive [16,18].

### 4.3. Statistical and In Silico Analysis

Associations between categorical variables—including FAP expression (negative vs. positive), pathological features, and treatment response—were assessed using the Chi-square (χ^2^) test.

Survival analyses for OS and DFS were carried out using Kaplan–Meier estimates and compared with the log-rank test. To determine independent prognostic factors, univariate and multivariate Cox proportional hazards regression models were applied, using the backward stepwise (Wald) elimination method.

All statistical analyses were conducted using SPSS^®^ software, version 28.0 (IBM Corp., Armonk, NY, USA).

To visualize TCGA data (Supplementary Figure S1) from kidney renal clear cell carcinoma (KIRC), we used GEPIA2 (http://gepia2.cancer-pku.cn, accessed on 8 October 2025); and R2: Genomics Analysis and Visualization Platform (http://r2.amc.nl, accessed on 8 October 2025).

## 5. Conclusions

This study reinforces the clinical significance of FAP expression in the tumor stroma of advanced ccRCC. We validated its association with adverse pathological features and demonstrated its potential as a prognostic biomarker of disease recurrence and overall survival. Importantly, we also identified a link between low stromal FAP expression and complete response to TKI therapy, highlighting its potential utility as a predictive biomarker. Taken together, these findings support the role of FAP as a marker of tumor aggressiveness, risk stratification and therapeutic response, and warrant further investigation into its integration into routine pathological assessment and clinical decision-making in ccRCC.

## Figures and Tables

**Table 1:** Pathological and clinical characteristics of patients at initial diagnosis.

Variables	*n* = 137
**Age**	
≤60 years	76
>60 years	61
**Gender**	
Male	97
Female	40
**Fuhrman grade**	
Low (G1–G2)	32
High (G3–G4)	105
**Diameter**	
≤8 cm	71
>8 cm	66
**Local invasion (pT)**	
Organ-confined (pT1–pT2)	37
Not confined (pT3–pT4)	100
**Lymph node invasion (N)**	
No	115
Yes	22
**Metastasis (M)**	
Metachronous	82
Synchronous	55
**NCCN 2010 Stage**	
Stage I–II	27
Stage III–IV	110
**ECOG** ^(^*^)^	
Preserved function (ECOG 0)	113
Reduced function (ECOG 1–2)	24
**IMDC** ^(^*^)^	
Favorable	59
Intermediate/Unfavorable	78

^(^*^)^ At initiation of TKI therapy.

**Table 2:** Predictive model (Cox regression) for 10-year disease-free survival (DFS) and 15-year overall survival (OS) in ccRCC patients. The model includes FAP expression (negative vs. positive) along with selected clinical, pathological, and treatment response variables. All variables were grouped and dichotomized as detailed in Table 1, and illustrated in Figure 2 and Figure 3. Hazard Ratios (HR) and corresponding 95% Confidence Intervals (CI) are reported. Variables identified as independent predictors through the backward stepwise (Wald) method are indicated in bold.

Cox Regression Model		10-Year Disease-Free Survival			15-Year Overall Survival		
	Variables	*p*	HR	95% CI	*p*	HR	95% CI
Univariate analysis	Sex	0.7	1.11	0.52–1.16	0.42	0.86	0.59–1.25
	Age	0.049	1.58	1.03–2.5	0.002	1.77	1.24–2.51
	Grade	<0.001	2.34	1.42–3.86	<0.001	2.1	1.38–3.11
	pT	0.001	2.32	1. 38–3.89	<0.001	2.07	1.38–3.08
	N	0.003	3.12	1.46–6.69	<0.001	2.86	1.79–4.57
	M	-	-	-	<0.001	3.06	2.13–4.38
	ECOG	-	-	-	0.01	1.78	1.15–2.75
	IMDC	-	-	-	<0.001	1.81	1.28–2.56
	MASS	-	-	-	<0.001	1.98	1.37–2.85
	RECIST	-	-	-	<0.001	3.1	1.7–5.66
	FAP	0.002	2.16	1.33–3.51	0.009	1.62	1.13–2.34
Multivariate analysis Final step of Wald method	Age	**0.002**	2.21	1.34–3.65	**0.008**	1.65	1.14–2.39
	Grade	**0.002**	2.4	1.39–4.15	-	-	-
	pT	**0.002**	2.45	1.41–4.26	**<0.001**	2.26	1.44–3.56
	N	-	-	-	**<0.001**	2.57	1.57–4.23
	M	-	-	-	**<0.001**	2.01	1.37–2.95
	MASS	**-**	-	-	**<0.001**	2.21	1.49–3.28

## Data Availability

The original contributions presented in this study are included in the article/Supplementary Materials. Further inquiries can be directed to the corresponding author.

## Supplementary Materials

The following supporting information can be downloaded at: https://www.mdpi.com/article/10.3390/ijms262211112/s1.
