# Supplementary material for: Fibroblast Activation Protein Alpha (FAP) Expression Is Associated with Disease Recurrence and Poor Response to Tyrosine Kinase Inhibitors in Advanced Clear Cell Renal Cell Carcinoma
Source: Int J Mol Sci. 2025 Nov 17;26(22):11112. doi: 10.3390/ijms262211112 (PMC12652626; doi:10.3390/ijms262211112)
Supplement: Supplementary file 1 [file ijms-26-11112-s001.zip › ijms-3899151-supplementary.pdf]

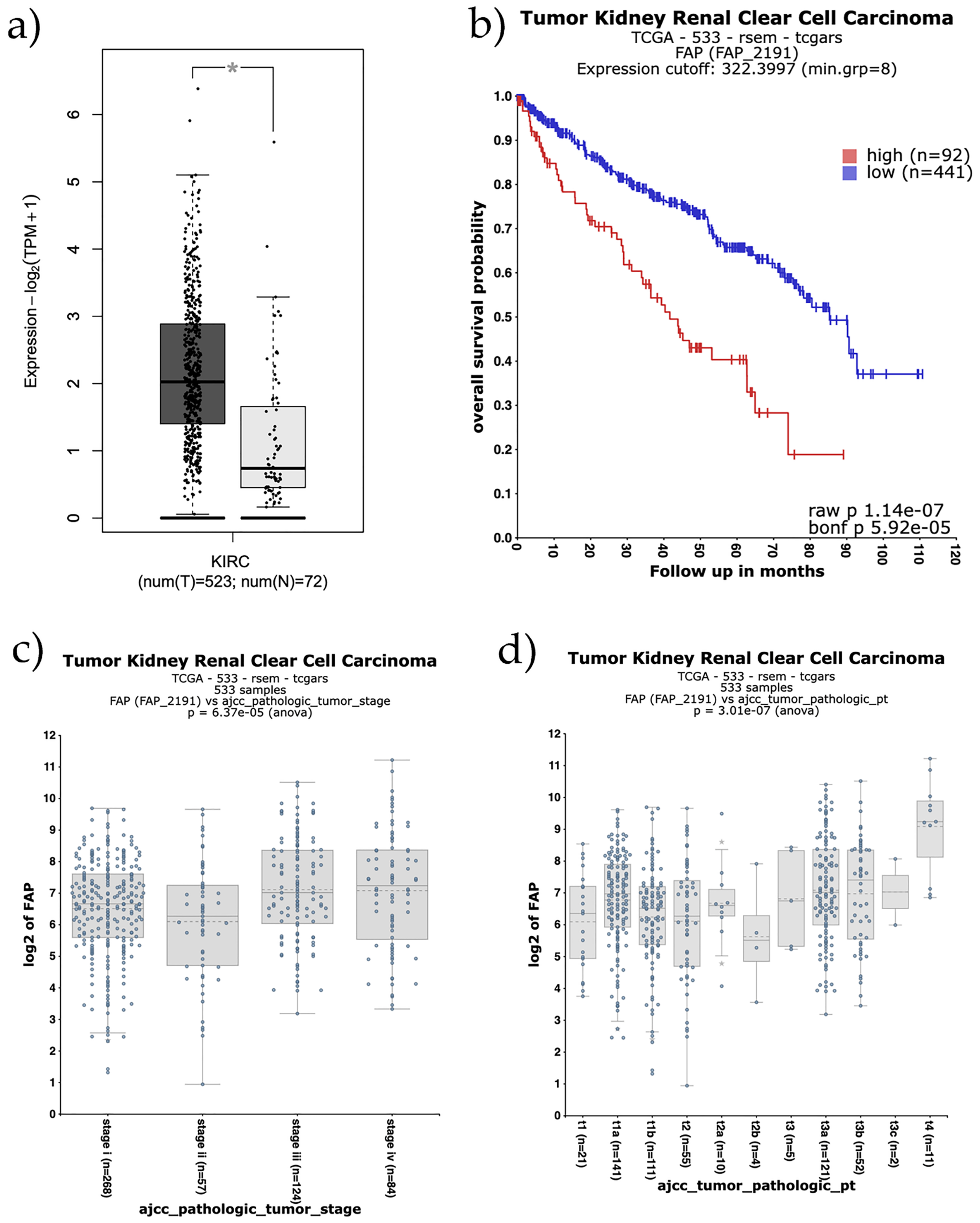

Supplementary Figure S1. In silico analysis of *FAP* mRNA expression in ccRCC. (**A**) Expression of *FAP* from TCGA data from ccRCC (KIRC) tumor (T, dark grey), and non tumor kidney tissue (N, light grey). (**B**) Overall survival (OS)

analysis according to high (n = 92) and low (n = 441) *FAP* expression. Kaplan–Meier curve showed that high FAP expression is associated with lower OS. (**C**) Higher pathologic stage primary tumors (AJCC 2010, Stage II–IV)) expressed higher *FAP* mRNA levels than lower ones (Stage I–II). (**D**) *FAP* expression was higher in non-organ confined (pT3 and pT4) primary tumors than in organ-confined ones (pT1 and pT2).
